# Congenital Mastoid Cholesteatoma With Giant Intracranial Extension: A Case Report

**DOI:** 10.7759/cureus.112838

**Published:** 2026-07-17

**Authors:** Nenad Novakovic, Dragan Zivojinovic, Svetozar Stankovic, Aleksandar Djurdjevic, Milan Lepic

**Affiliations:** 1 Clinic for Neurosurgery, Military Medical Academy, Belgrade, SRB; 2 Department of Neurosurgery, Medical Faculty of the Military Medical Academy, University of Defence, Belgrade, SRB; 3 Department of Pathology, Institute for Pathology and Forensic Medicine, Military Medical Academy, Belgrade, SRB

**Keywords:** case report, cholesteatoma, giant, intracranial cholesteatoma, primary mastoid cholesteatoma

## Abstract

Cholesteatomas are keratinizing cystic lesions of the temporal bone, most commonly involving the middle ear and mastoid. Intracranial extension is rare and usually associated with acquired disease, while giant supratentorial presentations are exceptional. We report a well-documented and neuroradiologically illustrated case of an exceptionally large congenital mastoid cholesteatoma with supratentorial intracranial extension. A 49-year-old woman presented with left parietal bone softening and recent ipsilateral facial sensory disturbance. MRI revealed a giant left temporoparietooccipital extra-axial mass (71 × 59 × 89 mm) with extensive calvarial destruction, extension from the mastoid, near-complete transverse sinus compression, and an 8-mm midline shift. She underwent a left parietooccipital craniotomy with complete evacuation of a tumor mass. Histopathology was consistent with cholesteatoma, and follow-up MRI at two months showed no residual or recurrent lesion. Clinically, she improved, with only mild residual conductive hearing loss and gradually resolving sensory symptoms. To our knowledge, no cases with comparable dimensions have been previously reported. This case complements the existing literature by representing the extreme upper end of the size spectrum of primary intracranial cholesteatomas.

## Introduction

Cholesteatomas are benign yet locally destructive lesions formed by the accumulation of desquamated keratin debris from stratified squamous epithelium surrounded by a fibrous matrix and are capable of progressive bone erosion and, rarely, intracranial extension. They most commonly arise within the pneumatized portions of the temporal bone, involving the middle ear and mastoid [1,2]. Based on their pathogenesis, cholesteatomas may be congenital or acquired. The term “primary mastoid cholesteatoma” refers to a congenital-type lesion with an intact tympanic membrane and no history of otorrhea, infection, or otologic surgery, irrespective of the extent of intracranial extension (criteria detailed in the Discussion). Congenital cholesteatomas are believed to result from abnormal development of the first branchial groove, leading to ectopic deposition of ectodermal tissue within the temporal bone, whereas intracranial localization is exceedingly rare, being reported in approximately 1% of cases in large series [3-5].

When intracranial extension does occur, it is most frequently associated with acquired cholesteatomas, and reports of giant lesions remain exceptional [4-6]. We present a case of a primary giant mastoid cholesteatoma with supratentorial propagation. To our knowledge, this represents one of the largest documented cases not only among congenital mastoid cholesteatomas but also within the broader group of congenital intracranial cholesteatomas.

## Case presentation

A 49-year-old female presented to the emergency department with a several-month history of a palpable area of bone softening in the left posterior parietal region and a recent history of altered sensation extending to the face, with no complaints of headache, hearing loss, vertigo, visual disturbances, or other sensory or motor symptoms. Initial CT revealed a massive extra-axial temporoparieto-occipital lesion displacing the tentorium and appearing as a supra- and infratentorial lesion. She was admitted to the neurosurgery clinic.

**Figure 1:**
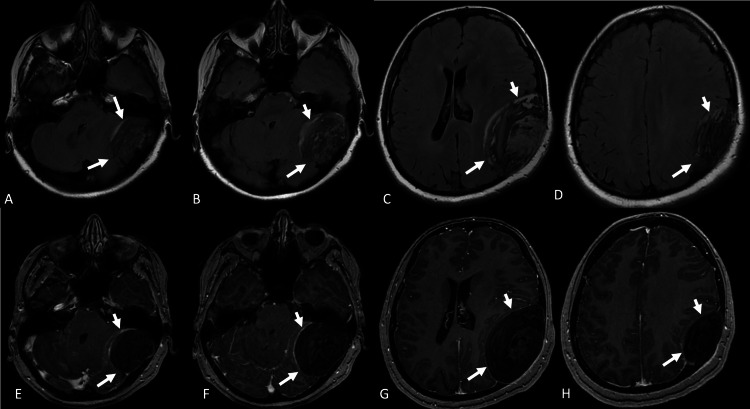
Preoperative axial T2 FLAIR (A-D) and post-contrast axial T1-weighted images (FSPGR BRAVO sequence) (E-H). White arrows point to the mass lesion. FLAIR, fluid-attenuated inversion recovery

**Figure 2:**
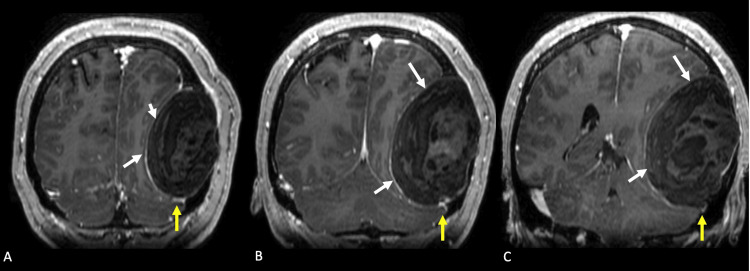
Coronal post-contrast T1-weighted images (A-C) demonstrating transverse sinus (yellow arrows) and suppression of the tentorium.

**Figure 3:**
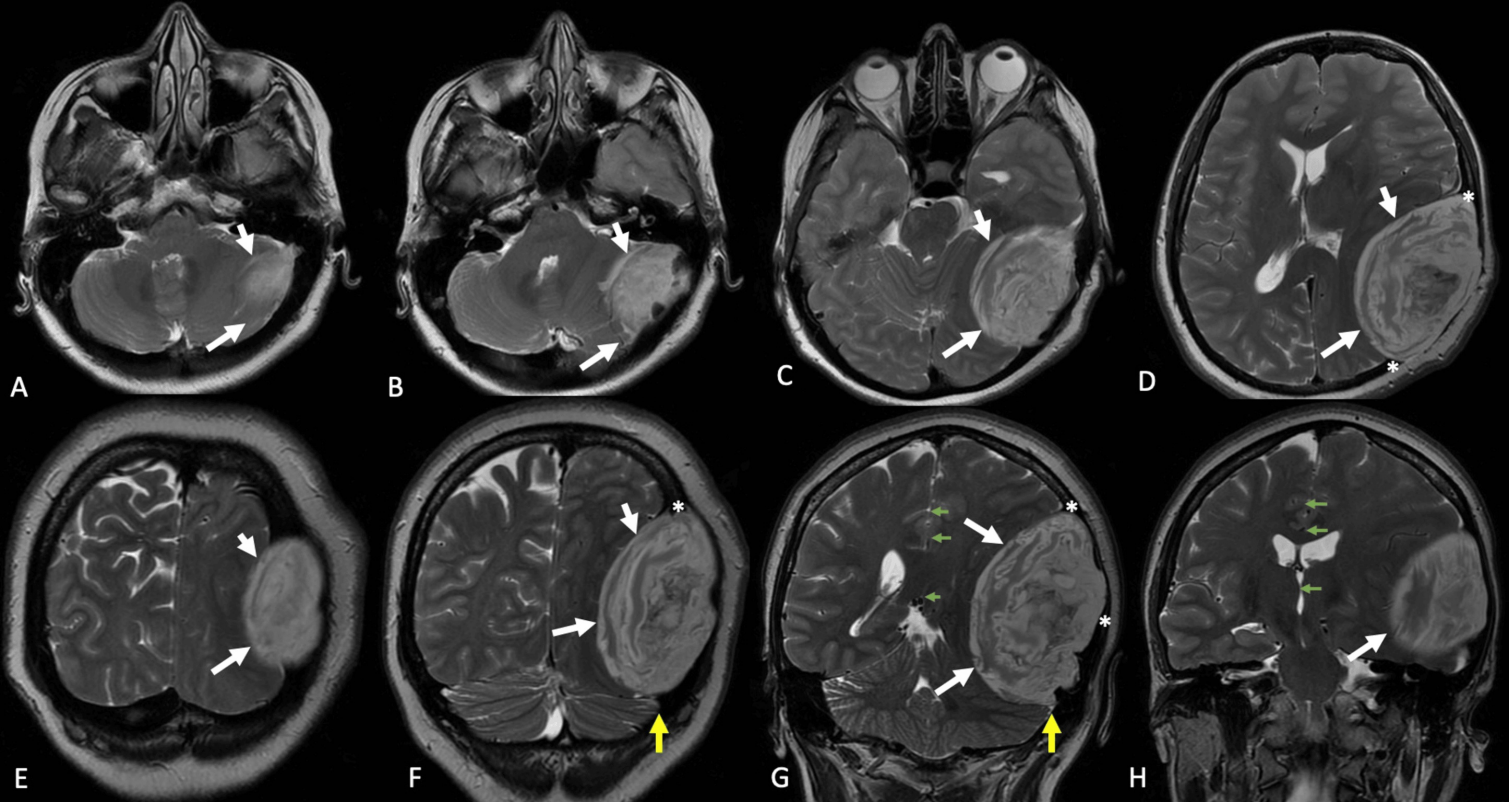
Preoperative axial (A-D) and coronal (E-H) T2 fast spin-echo MRI images demonstrate the lesion (white arrows) with a heterogeneous, partially laminated hyperintense signal and pronounced mass effect on the adjacent cerebral parenchyma. The asterisk indicates the bone defect, and green arrows indicate the midline shift.

**Figure 4:**
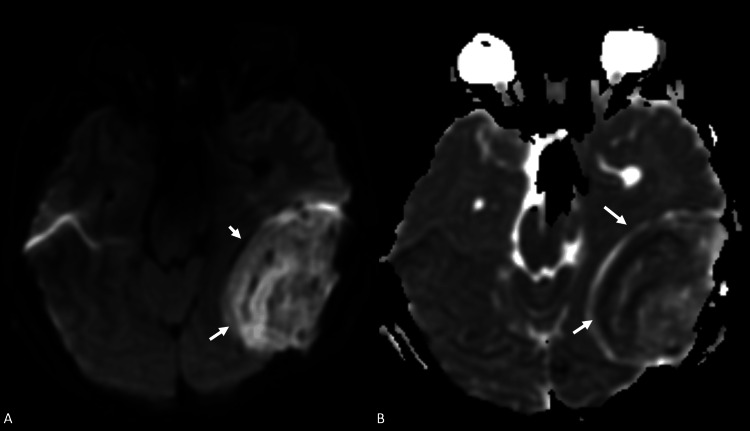
Preoperative DWI and ADC maps. DWI images (A) with the corresponding ADC map (B) demonstrate diffusion restriction within the lesion (white arrows). ADC, apparent diffusion coefficient; DWI, diffusion-weighted imaging

MRI (Figure 1A-1H, Figure 2A-2C, Figure 3A-3H) demonstrated a large oval extra-axial mass in the left temporoparieto-occipital region, measuring approximately 71 × 59 × 89 mm. The lesion had smooth, well-defined contours and a heterogeneous, partially laminated internal structure without post-contrast enhancement and with true diffusion restriction (Figure 4A, 4B). There was destruction of the squamous portions of the parietal and temporal bones, with extension into the mastoid segment of the left temporal bone. Mass effect was evident on the parietal, temporal, and occipital lobes, with compression of the left cerebellar hemisphere and downward displacement of the tentorium. The left transverse sinus was nearly completely compressed (Figure 2A-2C). The midline shift measured approximately 8 mm (Figure 3G, 3H), with signs of descending transtentorial herniation and rightward displacement of the midbrain. Both cerebellar tonsils extended 5 mm below the McRae line.

**Figure 5:**
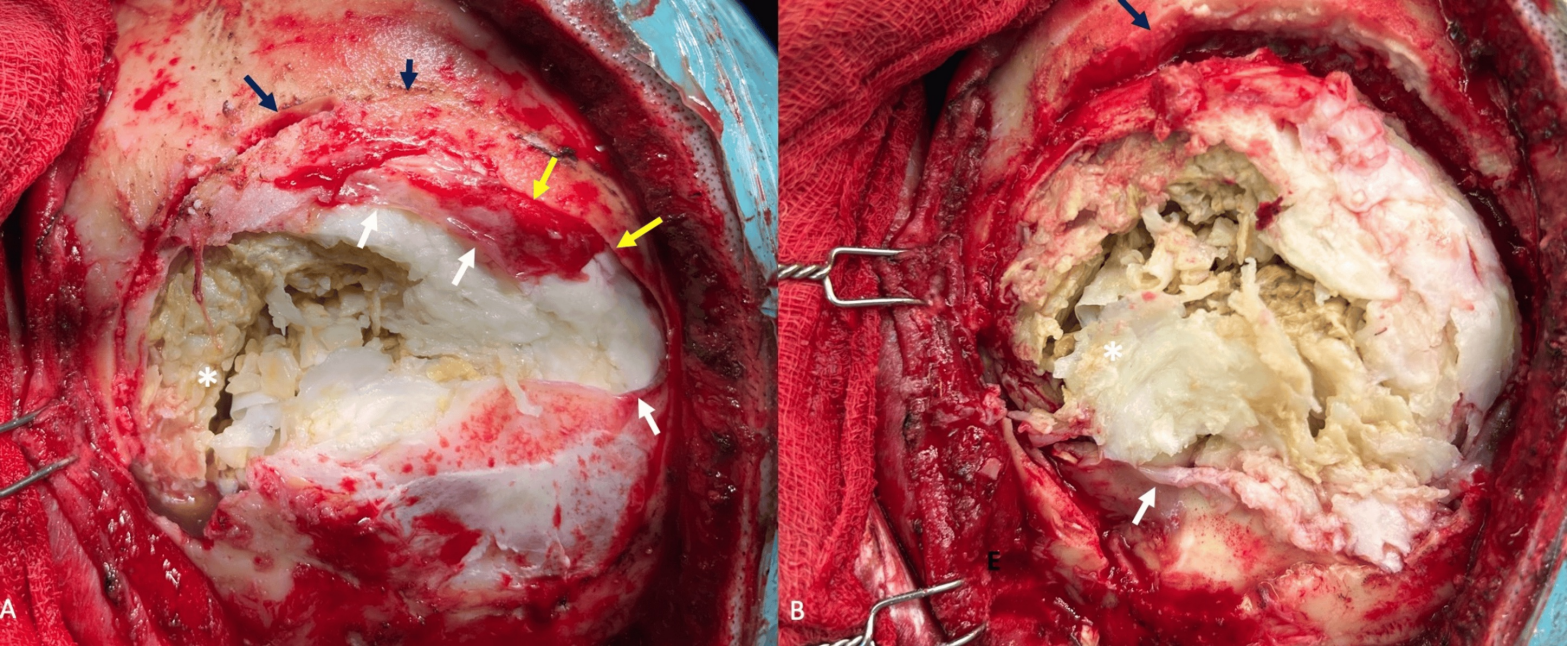
Intraoperative photographs. Operative stage following elevation of the skin flap and enlargement of the bony opening with the craniotome. Blue arrows indicate the craniotome trajectory before bone removal in panel A and after bone removal in panel B. White arrows indicate the membrane structure. Yellow arrows indicate the edge of the bone defect encountered after elevation of the skin flap. The asterisk indicates the lamellar keratin mass.

A left-sided parieto-occipital craniotomy was performed. Following a curvilinear skin incision, a bony defect filled with a firm, encapsulated, laminated keratinous matrix (white arrows in Figure 5A, 5B) was exposed (Figure 5A, 5B). After complete delineation of the margins of the osseous defect and gentle enlargement of the craniotomy, the majority of the matrix was successfully separated using blunt dissection. In the temporal region, focal dural adhesions were encountered; however, careful dissection was achieved without dural violation, and no dural reconstruction was required. Over the mastoid region, we identified opened mastoid air cells filled with keratinous matrix. The matrix was evacuated, and the opened mastoid was drilled to healthy bone and packed with bone wax.

**Figure 6:**
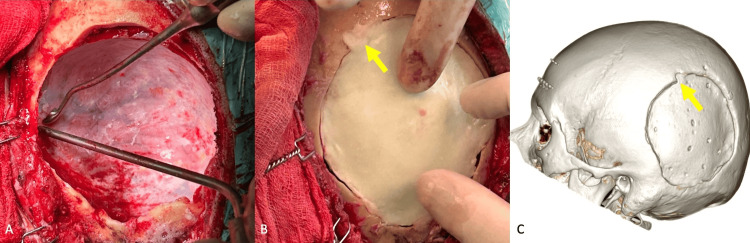
(A, B) Intraoperative photographs. (C) Postoperative multislice CT 3D reconstruction showing the cranioplasty. Yellow arrows indicate the same point on the polymethyl methacrylate vault for orientation.

After complete evacuation of the lesion, a hemispherical cavity was revealed, with no immediate intraoperative evidence of brain re-expansion or elasticity (Figure 6A). The cavity was irrigated and filled with saline solution, and cranioplasty was performed using polymethyl methacrylate (Figure 6B, 6C).

**Figure 7:**
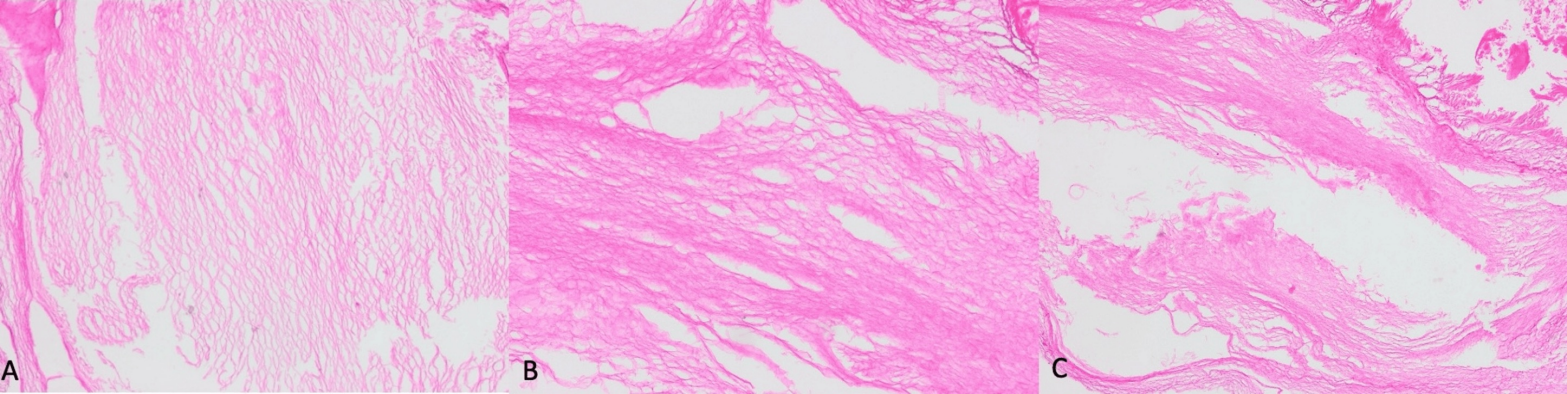
Histopathological examination demonstrating eosinophilic, acellular, lamellar keratin structures (A-C), consistent with the diagnosis of cholesteatoma. (A) H&E, ×40; (B) H&E, ×100; (C) H&E, ×40.

The early postoperative course was complicated by a sensation of ear blockage and transient anomic dysphasia, which resolved completely, with residual left-sided facial hypoesthesia. Postoperative CT demonstrated a small epidural hematoma at the site of the previous lesion, as well as a small subdural collection along the tentorium and falx; both resolved completely on follow-up imaging. Postoperative audiometry revealed mild conductive hearing loss, with an unremarkable otoscopic examination. Histopathology was suggestive of cholesteatoma (Figure 7A-7C). At the one-month follow-up, residual but less pronounced facial numbness was noted.

**Figure 8:**
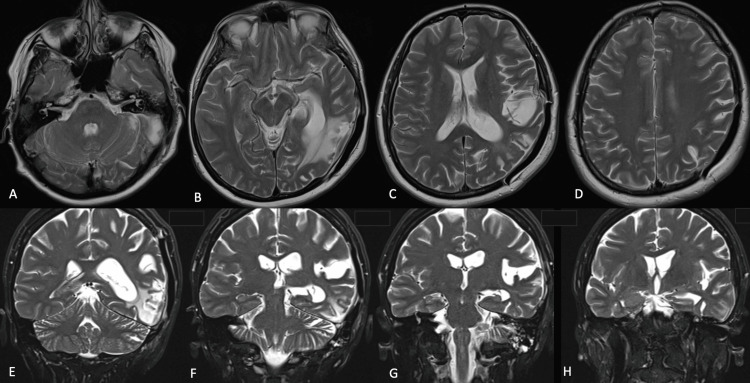
Postoperative T2 turbo spin-echo MRI images in the axial (A-D) and coronal (E-H) planes demonstrating a CSF-filled postoperative cavity in the left temporo-occipital region (B, C), surrounded by gliotic changes (B, E) and associated ex vacuo dilatation of the adjacent temporal horn (B, E). No residual or recurrent lesion is identified.

At two months postoperatively, MRI (Figure 8A-8H) demonstrated a CSF-filled postoperative cavity in the left temporo-occipital region (30 × 17 × 15 mm), involving the cortex and subcortical white matter, surrounded by a gliotic margin extending parietally and medially toward the periventricular white matter. No residual or recurrent lesion was identified. There was no residual or recurrent cholesteatoma in the temporal bone or operative field. The apical mastoid air cells were filled with fluid, while the mastoid was otherwise well pneumatized, with no pathological content in the antrum, attic, or tympanic cavity. Clinically, the patient reported further improvement of the parietal numbness, with gradual resolution of the left-sided ear fullness. She remains under regular otolaryngological follow-up, with the most recent audiological evaluation demonstrating residual conductive hearing loss and bilateral type A tympanograms. The patient remained clinically stable at the six-month follow-up.

## Discussion

This report highlights an exceptionally large, neuroradiologically well-documented giant congenital mastoid cholesteatoma with supratentorial intracranial extension and extensive osseous destruction, managed through a neurosurgical corridor with effective matrix evacuation and reconstruction.

“Cholesteatoma” is used inconsistently across the otologic, skull-base, and neurosurgical literature and sometimes overlaps with terms such as “epidermoid” or “epidermal inclusion cyst,” depending on the anatomic site and discipline [1-3]. The term “primary” is also used inconsistently, sometimes to denote a congenital-type cholesteatoma [7,8], yet elsewhere to describe a variant of acquired cholesteatoma (“primary type”) [3]. For temporal bone disease, classification by pathogenesis (congenital vs acquired) and by site of origin is clinically meaningful [1]. Primary mastoid cholesteatoma is defined [7] using strict criteria: (1) it must fulfill the features of congenital cholesteatoma, an intact tympanic membrane, no history of otorrhea or infection, and no prior otologic surgery; and (2) there must be no involvement of the middle ear, attic, or aditus. In our case, the absence of middle ear, attic, or aditus involvement on follow-up imaging, together with the clinical context, supports classification within the spectrum of a primary congenital mastoid cholesteatoma rather than a typical acquired middle ear cholesteatoma with secondary mastoid spread.

The radiologic differential diagnosis for this type of lesion included chronic subdural collection, cystic or necrotic metastasis, meningioma, abscess, and dermoid and epidermoid cysts. The intradiploic epidermoid cyst represented the most challenging differential diagnosis because it shares similar radiologic features. Distinction therefore relies on anatomic findings: imaging and intraoperative exploration demonstrated opened mastoid air cells filled with keratinous matrix in direct continuity with the intracranial mass, with concentric keratin layers originating from the mastoid. However, mastoid involvement does not definitively establish that the lesion originated there rather than secondarily invading the mastoid, and the overlap in imaging findings means that the site of origin cannot be established with absolute certainty from imaging or intraoperative findings alone. While secondary erosion of a primary calvarial epidermoid into an adjacent pneumatized mastoid has been described [9], such cases typically present with a dominant extracranial diploic component and a discrete bony erosion pathway into the mastoid, rather than the pattern seen here. We therefore favor a primary mastoid origin based on the demonstrated anatomic findings and fulfillment of the abovementioned clinical criteria, while acknowledging that a giant intradiploic epidermoid with secondary mastoid extension remains a differential diagnosis that cannot be entirely excluded.

Intracranial extension is uncommon overall. Large series suggest rates of around 1% [3,4,6], but when it occurs, it is most often described in the setting of acquired aural cholesteatoma. Horn’s work is particularly relevant because it details how acquired cholesteatoma may enter the middle or posterior cranial fossa through characteristic temporal bone pathways and emphasizes the skull base implications of this disease [3]. Well-illustrated “giant” intracranial cholesteatomas remain rare despite the long history of case reports in the literature [10]. Among contemporary reports, one described a massive temporal lobe cholesteatoma measuring up to 55 mm [4], while another documented the first lesion with combined supratentorial and cervical extension, measuring 80 × 54 mm [11]. Other “giant” presentations include extensive skull base or petrous apex disease, sometimes complicated by infection [5], or arising in atypical cranial locations [6]. In a focused review of primary mastoid cholesteatoma, the authors analyzed 25 cases in which intracranial propagation was confined to the posterior fossa, and none required craniotomy [8]. This review highlights how unusual a supratentorial presentation is within this subgroup. Similarly, other authors reviewed nine extratemporal intracranial cholesteatomas and identified two “massive” cases, but both had limited infratentorial extension [12].

## Conclusions

With preoperative dimensions of 71 × 59 × 89 mm and an almost entirely intracranial, exclusively supratentorial extension, the present lesion represents, to our knowledge, one of the largest reported cases within the spectrum of giant congenital mastoid cholesteatoma. A primary mastoid origin is favored over a giant intradiploic epidermoid with secondary mastoid extension based on the anatomic and radiologic findings discussed above, although the latter cannot be entirely excluded. This case extends the reported size spectrum of primary mastoid cholesteatoma and highlights that such giant, purely supratentorial presentations should be considered in the differential diagnosis of large extra-axial calvarial lesions.
